# Acquisition of non-contrastive focus in Russian by adult English-dominant bilinguals

**DOI:** 10.3389/fpsyg.2024.1363980

**Published:** 2024-12-24

**Authors:** Tatiana Luchkina, Tania Ionin, Maria Goldshtein

**Affiliations:** ^1^Linguistics Department, Stony Brook University, Stony Brook, NY, United States; ^2^Linguistics Department, University of Illinois at Urbana-Champaign, Urbana, IL, United States; ^3^Ira A. Fulton Schools of Engineering, Arizona State University, Tempe, AZ, United States

**Keywords:** focus, information structure, prosody, constituent order, Russian

## Abstract

This study investigates the acquisition of sentence focus in Russian by adult English-Russian bilinguals, while paying special attention to the relative contribution of constituent order and prosodic expression. It aims to understand how these factors influence perceived word-level prominence and focus assignment during listening.

We present results of two listening tasks designed to examine the influence of pitch cues and constituent order on perceived word prominence (Experiment 1) and focus assignment (Experiment 2) during the auditory comprehension of SV[O]_F_ and OV[S]_F_ sentences in Russian. Our findings reveal an asymmetric pattern: monolingual speakers, as a baseline, tend to perceive the nuclear pitch-accented object as more prominent, particularly in the SVO order, whereas bilinguals appear to be less sensitive to the constituent order distinction.

Additionally, baseline speakers consistently assign focus to the sentence-final nuclear pitch-accented noun regardless of constituent order. In contrast, bilinguals demonstrate a preference for assigning focus to the sentence-final nuclear-accented object, rather than the sentence-final nuclear-accented subject. A proficiency effect emerged indicative of a more target-like performance among bilinguals with greater proficiency in Russian.

## 1 Introduction

The present study critically evaluates the ability of adult English-Russian bilinguals to infer sentence focus in Russian, a free word order language, in both canonically ordered SVO sentences and non-canonically ordered OVS sentences, during auditory sentence comprehension.

Similar to other languages with pitch accents, Russian exhibits prosodic effects tied to the information status of referents, which is reflected in pitch accent patterns at the phrasal level. This includes emphasizing new, focused information while de-emphasizing given information (Neeleman and Titov, 2009; Jasinskaja, 2016).

The Nuclear Stress Rule (NSR; Chomsky and Halle, 1968) establishes that the main phrasal prominence, or nuclear pitch accent, is placed at the rightmost prosodic domain boundary. In both Russian and English, Intonational Phrases (IPs) define the prosodic domain within which the NSR operates. Russian shares similarities with English in that focusing a non-IP-final word shifts the nuclear pitch accent to a non-phrase-final position to align with the focused constituent. However, unlike English, Russian uses overt case morphology and alters the order of sentence constituents to convey information status and relative prominence (Bailyn, 1995; Kallestinova, 2007; Slioussar, 2007).

Consider the example provided in (1); the subject question in (1a) can receive a response with non-canonical OVS order, shown in (1b) with the focused subject noun “lisa” (eng.: “fox.NOM”) positioned at the end of the sentence and aligned with the nuclear pitch accent sentence-finally. A similar pattern emerges in the object question in (2a), which can receive a response in the baseline SVO order, with a sentence-final nuclear pitch accent (2b)^1^. Examples 3 and 4 illustrate the same question-answer pairs in English; since OVS order is not possible in English, both (3b) and (4b) have SVO order, but the nuclear pitch accent is aligned with either the subject or the object, depending on the question type.

Here and below, caps in example sentences indicate the nuclear accent and _F_ represents the focus. 
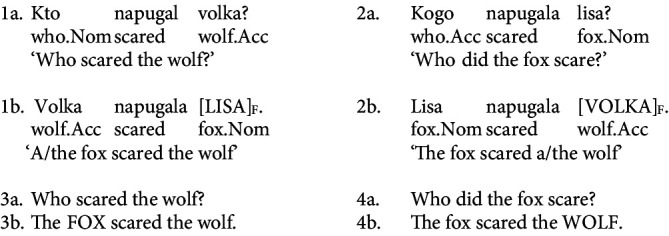


Example 1 demonstrates that in Russian, the focal reading of a sentence element allows for its relocation to the final position in the utterance which serves as the primary landing site for the main phrasal prominence, i.e., the nuclear pitch accent. While baseline monolingual speakers of Russian demonstrate sensitivity to focus-driven constituent order alternations (Laleko, 2022) and exhibit variability in prosodic expression linked to information status distinctions (Luchkina and Cole, 2021), heritage speakers and second language (L2) learners of Russian have been found to display non-native-like patterns of acceptability for non-canonical orders (Laleko, 2022; Ionin et al., 2023a; Ionin et al., 2023c).

This observation suggests that the concurrent use of constituent order and prosody in the expression of sentence focus may pose a challenge for adult L2 speakers of Russian. Acquisition challenges may stem from learners encountering difficulties in identifying sentence focus through prosodic cues, or in integrating word order and acoustic-prosodic expression with the discourse context (Ionin et al., 2023a).

The Interface Hypothesis for second language acquisition (Sorace and Filiaci, 2006) predicts increased complexity and resistance in acquiring properties that require the integration of language-internal and language-external domains, including syntax and information structure.

Interface phenomena investigated by Sorace (2011), along with much subsequent research, exhibited optionality in the interlanguage (IL) grammars of adult L2 learners, which contrasts with the more consistent grammars of native (baseline) speakers. For instance, Sorace (2007) examined the use of overt and null subjects by highly proficient L1 English learners of L2 Italian. In Italian, the use of null subjects is determined by the information status of the subject (new vs. given), with discourse-given subjects typically being null. Sorace reported residual optionality (i.e., the use of both overt and null subjects) among the tested L2 learners in contexts where native speakers consistently opted for null subjects. Sorace attributed this optionality in the use of null subjects by L1 English learners of L2 Italian to the complex nature of the interface between syntax and discourse that is inherent in this aspect of Italian syntax.

In the context of the present study, information structure in L2 Russian requires the simultaneous use of distinct target language properties, including constituent order and prosodic expression. The associated acquisition challenges documented in earlier research may therefore stem from learners’ difficulties in identifying sentence focus through prosodic cues, or from the integration of word order and acoustic-prosodic expression with the discourse context, as predicted by the Interface Hypothesis.

The present study assesses these possibilities by comparing monolingual speakers of English and Russian with adult English-Russian bilinguals whose dominant language is English. These speaker groups are compared on their perception of the main phrasal prominence in sentences such as (1b) and (2b) and further, on concurrent use of constituent order and prosodic expression as cues to focus assignment during listening.

## 2 Expression of sentence focus

### 2.1 Pitch accenting

In the influential research by Chafe (1976), focus is defined as an intrinsic attribute of the utterance information structure. In the present study, we use the term “focus” to signify newly introduced information within a sentence that is expected to be the primary point of interest for the listener or reader [see Cruschina (2022) for more discussion].

In pitch accenting languages, including Russian and English, focal information tends to be prosodically distinct due to relative prosodic augmentation of the sentence focus in combination with partial reduction of prominence of non-focal, given information. Extensive foundational research on spoken English has established a clear link between heightened information emphasis, often attributed to focal status, and prosodic prominence (Beckman and Pierrehumbert, 1986; Selkirk, 1995; Ladd, 2008; Büring, 2009; Wagner et al., 2010; Cole, 2015; Bishop et al., 2020).

Sentence focus frequently exhibits a distinct prosodic expression, thus rendering it prosodically prominent, as discussed in the works of Selkirk (1995), Ladd (2008), Büring (2009), Calhoun (2010), and Bishop et al. (2020). In English, focus prominence results from distinctive pitch accenting patterns linked to the relative information prominence of a word. When a word holds focal status, it is assigned a nuclear pitch accent, effectively linked to the most perceptually salient prosodic event within a larger domain, such as an IP. The form of the pitch contour indicating focus or discourse-new information status is informed by the specific pitch accent type, such as H* (Pierrehumbert and Hirschberg, 1990; Beckman et al., 2005).^2^

Perception-production studies by Gussenhoven and Rietveld (1988), Xu and Xu (2005), Breen et al. (2010), and Bishop et al. (2020) reported significant contribution of the local pitch maxima, the speed of pitch rise and the size of pitch excursion over the focused word to acoustic-prosodic expression of focus in English. In perceptual terms, the augmented prosodic expression translates into heightened prosodic prominence of the focal material (Xu and Xu, 2005; Cole, 2015), which may further translate into variable degrees of perceived information prominence by linguistically naïve listeners (Breen et al., 2010).

Given the various mechanisms that contribute to the focus prominence, studies examining the production and perception of sentence focus reveal inherent variability in how speakers express it orally, as well as in how listeners perceive it (Breen et al., 2010; Takahashi et al., 2018).

The work of Breen et al. (2010) presents an illustrative perception-production study of English focus. In their comprehensive analysis of the acoustic-prosodic focus correlates, the authors emphasized the crucial role of several acoustic parameters, including pitch, loudness, and segmental length in distinguishing the focused element from the rest of the sentence. In a series of discriminant function focus identification analyses, prosodic expression helped determine the location of the focused word in test sentences but proved insufficient to discriminate between contrastive (LH*) vs. non-contrastive focus (H*) or determine the size of the focus domain (broad vs. narrow). Linguistically naïve listeners tested by Breen et al. (2010) were highly successful at locating the sentence focus (10/10 succeeded) but only moderately successful at identifying the focus type (contrastive vs. non-contrastive, 6/10 succeeded) or the focus domain size^3^ (8/10 succeeded).

The same study by Breen and colleagues analyzed read production-perception data from 13 unique sets of speakers. In each speaker pair, partner 1 read a target sentence and partner 2 selected one of the seven questions for which participant 1’s production served as the most plausible answer.^4^ The authors reported an overall accuracy of 55%, which was above chance given the large number of context options available to the listeners. About half (46%) of Breen et al.’s (2010) participants achieved above chance accuracy at identifying wide focus, and 70% of the participants were above chance at identifying narrow object focus phrase-finally.

The prosodic correlates of sentence focus in Russian have been investigated by Bryzgunova (1980) and Zybatow and Mehlhorn (2000). In a more recent review by Jasinskaja (2016), the prosodic analysis of Russian focus is grounded in a detailed examination of intonational patterns, pitch accents, and their interaction with syntactic structure and discourse context. Jasinskaja (2016) bases her prosodic analysis of Russian focus on Bryzgunova’s (1980) pitch (intonational) contour classification, originally developed for categorizing “neutral” and “non-neutral” intonation patterns in Russian. Using Bryzgunova’s terminology, the *neutral intonational contour* pertains to the SVO sentence pattern with new information focus positioned toward the end of the phrase or aligned with its rightmost edge. In terms of prosody, sentences aligns clause-final new information focus can feature several down-stepped pre-nuclear pitch accents on each pre-focal word. The H tone of the HL* bitonal nuclear accent aligns with the pretonic syllable of the focused word exponent, leading to a drop in pitch over the stressed syllable.

Word-level augmented prosodic expression of non-contrastive new information foci was identified as a reliable predictor or perceived information prominence by adult Russian listeners by Luchkina and Cole (2021). This effect was further amplified by variations in word order, a topic we explore next.

### 2.2 Word order

Due to the relatively free constituent order in the Russian language, Kallestinova (2007) and more recent studies by Luchkina and Cole (2021), Ionin et al. (2023a), and Laleko (2022, 2024) have investigated the role of word order in signaling sentence focus. This research has demonstrated that the SVO and OVS orders in Russian correspond to distinct configurations in terms of information structure. In the baseline SVO order, the subject is generally assumed to be part of the ongoing discourse, while the object is considered new information and is in focus. Conversely, with OVS order, the object is established in the discourse, i.e., is topical, while the subject takes center stage in the listener’s attention, i.e., is in focus.

SVO [as in (2b)], which is typically seen as the default word order (Bivon, 1971), can be adjusted prosodically to suit different IS scenarios (Laleko, 2024). In contrast, OVS [as in (1b)] appears more marked (Sekerina, 1999) and necessitates an interpretative license (Titov, 2017). According to Kallestinova’s experimental research on constituent orders in Russian, speakers use OVS when they want to emphasize the subject, but not in other contexts (Kallestinova, 2007). This suggests that the limited applicability of non-standard word orders renders them less amenable to prosodic adjustments Luchkina et al., (in prep). Overall, the experimental evidence points to a distinct interplay between word order and prosody in Russian, particularly in scenarios involving subject and object focus.

**Figure 1:**
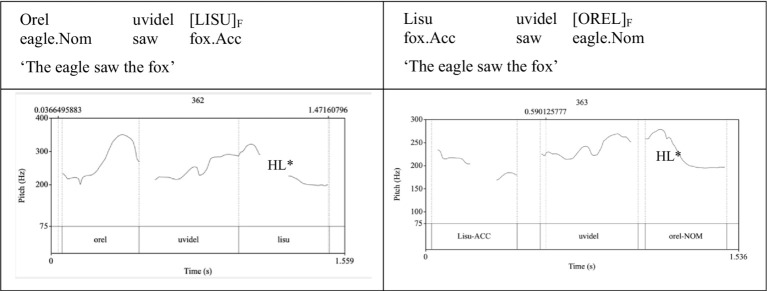
Illustrative pitch tracks of an SVO sentence (left) and an OVS sentence (right) ‘The eagle saw the fox’ with HL* clause-final nuclear pitch.

Figure 1 offers illustrative pitch contours associated with baseline SVO order in and the subject-final OVS order. Both pitch tracks illustrate clause-final placement of the nuclear pitch prominence.

## 3 L2 acquisition of sentence focus

While constituent order and prosodic cues are generally reliable indicators of distinctions in information structure for native speakers, they pose a recognized difficulty in the acquisition for adult L2 learners and heritage language speakers.

### 3.1 Known acquisition challenges

One potential source of difficulty in identifying focus based on auditory cues is the subtle and variable nature of the nuclear pitch prominence, which serves as the acoustic-prosodic expression of new information focus. Although nuclear pitch accent is often cited as the most reliable cue to sentence-level prominence and focus (e.g., Gussenhoven, 2004), no single acoustic correlate of nuclear pitch prominence has been established for English, Spanish, or Russian, such that it would enable identification of the prominent word directly from quantitative acoustic measurements, without an auditory analysis (Beckman, 1996; Fletcher and Evans, 2002).

The probabilistic nature of focus expression during speech underlies a great deal of individual variability in focus perception and production. A recent study by Takahashi et al. (2018) compared native English speakers and L1 Mandarin L2 English learners on the production and perception of narrow contrastive focus in English. When examining the use of acoustic-prosodic expression in relation to the focused constituent, it was observed that not all baseline speakers chose to produce a nuclear pitch accent in the vicinity of the sentence focus. Furthermore, expressing focus through prosody during production did not determine whether the same speaker relied on acoustic-prosodic cues for auditory focus identification. Takahashi et al. reported a similar ‘disconnect’ between the production and perception of English contrastive focus in a group of proficient L1 Mandarin L2 English speakers. These findings highlight the inherent inter-speaker variability and the probabilistic nature of the prosodic cues used to convey focus in the context of L1-L2 English.

Another challenge may arise from the relatively greater complexity of focus expression, which involves bridging multiple domains, including prosody, syntax, and information structure.

The Interface Hypothesis (IH), formulated by Sorace and Filiaci (2006), provides a theoretical framework that underscores the challenges associated with acquiring phenomena at the intersection of a language-internal syntax domain and language-external phenomena, including information structure. Due to the greater underlying complexity of interface phenomena, the targeted interface structures resist acquisition, even in IL grammars of learners at advanced proficiency levels. In particular, Sorace (2011) proposed that the acquisition of external interface phenomena is linked to instances of optionality within the target grammar, as well as “protracted indeterminacy” found even in near-native L2 learners (2011, p. 5). This stands in contrast to structures that are purely language-internal and, therefore, more readily acquirable.

Experimental evidence supporting the IH emphasizes the transfer of focus marking strategies from the native language, as shown in studies by Hertel (2003), Fruit (2006), and Ortega-Llebaria and Colantoni (2014). To illustrate, Fruit (2006) examined how Brazilian Portuguese (BP) speakers of varying English proficiency levels interpreted different focus structures in both BP and English. Fruit observed that even the L2 speakers considered to have achieved near-native proficiency in the TL exhibited L1 influence in their selection of constituent order and accent placement. For example, some participants showed a preference for sentence inversion in cases of narrow focus, which deviated from the standard SVO order used in combination with prosodic emphasis on the focused word in the TL. Fruit concluded that the interface between syntax and information structure presents a challenge in acquisition, even for L2 learners whose TL syntax is generally similar to the native language. Fruit identified L1-biased optionality and transfer from L1 to L2 as probable factors contributing to the variable performance observed among the tested participants, even among those who otherwise exhibited convergence with the TL syntax.

In a similar vein, Ortega-Llebaria and Colantoni (2014) found that learners’ focus marking patterns in their native language may result in lasting transfer effects when acquiring an L2, regardless of proficiency level. The authors examined how native Spanish and native Mandarin speakers learning English as an L2 perceived sentence focus. Both groups were tasked with identifying the location of the word in contrastive focus in sentences presented with or without context. The study found that native Mandarin speakers demonstrated a high level of accuracy, closely resembling native English speakers, even though their overall proficiency in English was lower than that of native Spanish speakers. This accuracy likely stemmed from positive transfer from their native language, which, like English, employs prosodic prominence to convey contrastive focus. In contrast, native Spanish speakers, despite their higher TL proficiency, showed noticeably lower accuracy. This discrepancy may be attributed to the Spanish tendency to use word order to position the focused element at the end of the sentence, where it receives the nuclear pitch accent. As expected, the accuracy of L1 Spanish speakers was greater when the focused element was the post-verbal object compared to when it was the pre-verbal subject, underscoring the strong influence of the L1 focus-marking strategies.

Notably, several experimental investigations of production and perception of sentence focus brought forward evidence supporting that L2 focus is acquirable and that successful acquisition critically depends on the proficiency in the TL. To illustrate, a production study on inverted VS order in the expression of Spanish information structure conducted by Hertel (2003) with L1 English L2 Spanish learners revealed a presence of L1 transfer from English, particularly at lower and intermediate levels of TL proficiency. An emerging sensitivity to discourse factors, including focus, was observed in advanced-level learners who demonstrated a native-like preference for the *VS* constituent order used to signal subject focus.

In summary, challenges in acquiring L2 focus may stem from differences in linguistic means used as focus cues between the speakers’ L1 and the TL (e.g., information structure primarily interfaces with constituent order in Spanish but with phrasal prosody in English). As predicted by the Interface Hypothesis, when the domain in question—focus—intersects language-internal and language-external elements, that domain becomes vulnerable and resistant to acquisition. This intersection complicates the learning process, making it more challenging to fully acquire the relevant interface structures.

### 3.2 Evidence from L2 Russian

In recent years, several experimental investigations have focused on the acquisition of the information structure and its effects on constituent order in Russian. Ionin et al. (2023a) and Laleko (2022) both conducted acceptability judgment studies, where English-dominant English-Russian bilinguals evaluated the acceptability of baseline SVO and inverted OVS stimuli sentences, considering the focal reading of one of the nominal constituents.

Ionin et al. (2023a) utilized pre-recorded auditory SV[O]_F_ and OV[S]_F_ test sentences featuring narrow focus clause-finally. Acceptability patterns varied among bilinguals, with heritage speakers, but not adult L2 learners, interpreting the OVS order as a means of signaling subject focus. Laleko (2022) reported similar results using written stimuli sentences. Laleko’s study, similarly, found that heritage bilingual speakers with higher Russian proficiency, but not adult Russian L2ers, succeeded at accepting the subject-final order in transitive OV[S]_F_ sentences with subject focus.

Laleko (2022) extended her investigation into the information structure domain in heritage Russian by assessing the acceptability of pre-recorded SVO and OVS sentences. Focus in these sentences was marked either through prosodic cues or constituent reordering. An asymmetry surfaced, where baseline monolingual speakers exhibited no preference for either focus marking strategy. In contrast, heritage speakers clearly favored nuclear pitch accenting of the focused word *in situ*, rendering constituent reordering redundant. The same study reported that heritage speakers over-accepted phrase-final nuclear pitch prominence under narrow subject focus in the SVO order. In contrast, under object focus, they correctly rejected infelicitous placement of the nuclear accent in the sentence-initial position. Laleko (2022) interpreted these findings as evidence of partial “neutralization in prosodic patterns” by heritage Russian speakers (p. 16).

Recent evidence supporting on-target perception of prosodic cues in relation to contrastive sentence focus in Russian was presented by Ionin et al. (2023b) who tested contrastive focus (CF) identification in SV[O]_CF_, S[O]_CF_V, and [S]_CF_VO experimental sentences, preceded by a one-sentence discourse context. The study found that adult L2 learners of Russian successfully identified the word in contrastive focus, regardless of whether it occurred sentence-finally (SV[O]_CF_) or elsewhere (e.g., S[O]_CF_V).

Experiment 2 in Ionin et al. (2023b) assessed focus identification during silent reading, requiring listeners to rely solely on context cues, and during listening, where the word in focus was made prosodically prominent. In the listening phase, both felicitous and non-felicitous contexts were examined to measure listeners’ ability to determine the location of nuclear pitch prominence in the absence of supportive context cues. The study reported a notably accurate performance from 26 adult English-Russian bilinguals, with above 90% accuracy during silent reading and listening. During listening, identification accuracy remained well above chance even when the target sentences were presented along with non-felicitous contexts (the context sentence set a non-nuclear accented noun in focus). This reveals listeners’ sensitivity to the prosodic cues in expression of contrastive focus under various constituent orders and phrasal locations. Ionin et al. (2023b) reported that the participants’ TL proficiency served as a crucial predictor of accurate contrastive focus identification during listening.

Contrary to Ionin et al. (2023b), a related investigation of non-contrastive focus in Russian, by Luchkina et al. (in press), reported considerably more indeterminacy on part of both native Russian listeners and adult English-Russian bilinguals when these groups were tested on auditory comprehension of SV[O]_F_ and OV[S]_F_ sentences featuring an instance of non-contrastive new information focus clause-finally. Near-ceiling accuracy was achieved during the silent reading task. During listening, participants had to identify the most prosodically prominent word in the test sentences presented with context. The study reported a 57% rate of perceived nuclear prominence in felicitous question-answer pairs (object question followed by an SV[O]_F_ target) and a low 28% accuracy on non-felicitous question-answer pairs (object question followed by an OV[S]_F_ target) in their data from English-Russian bilinguals. These findings reveal that bilinguals were more likely to identify the nuclear-accented word as prominent when it was in focus. A follow-up analysis, in which participants’ TL proficiency was considered, revealed robust prominence identification in congruent question-answer pairs but a proficiency-dependent outcome for non-congruent, illicit question-answer pairings. The context felicity effect and its interaction with TL proficiency jointly point to less proficient bilingual listeners relying on context cues, rather than prosodic cues, for focus identification.

In summary, prior experimental investigations have emphasized the complexity of acquiring sentence focus in Russian as the TL. This complexity arises from the involvement, on one hand, of distinct language-internal means of signaling focus in spoken language use, including prosodic cues and constituent order, and on the other, coordinating these mechanisms with the language-external domain of information structure.

## 4 The present study

In this study, we further explore the perception of nuclear pitch prominence using the test stimuli from Ionin et al. (2023a) and Luchkina et al. (in press). We present novel perception data from monolingual reference groups of Russian and English listeners, and English-dominant bilinguals. Considering the TL proficiency effect on the perceptual judgments of sentence prominence reported in the earlier related work, this investigation focuses on English-Russian bilinguals whose scores from an independent proficiency measure (cloze deletion test) substantiate a significant level of proficiency in Russian.

The present study is guided by two primary objectives. The first objective is to investigate perceived word-level prominence based on (1) tonal cues associated with the auditory expression of sentence focus in the languages spoken by the population of interest: English (dominant) and Russian (non-dominant) bilinguals and (2) linear order of the sentence constituents in Russian. The second objective of this study is to empirically evaluate how both constituent order and tonal cues linked to nuclear pitch prominence are employed concurrently in Russian during focus assignment, a task undertaken by native speakers and English-Russian bilinguals.

We begin, in Experiment 1, by assessing the perceptual weight of auditory cues to phrase-final nuclear pitch prominence across three speaker groups—two monolingual reference groups of English and Russian speakers, and a test group consisting of English-Russian bilinguals. This analysis aids in assessing whether the non-target-like performance exhibited by bilingual speakers, as noted in previous studies (Ionin et al., 2023a; Luchkina et al. in press), can be linked to the perception of tonal cues to word-level prosodic prominence in Russian by English-dominant bilinguals.

To this end, in Experiment 1, we assess whether proficient English-Russian bilinguals demonstrate a target-like use of prosodic cues to phrase-final nuclear pitch prominence in Russian SVO and OVS stimulus sentences. The following research questions are addressed: (1) What cues underly the percept of prosodic prominence in simple transitive Russian sentences? (2) Do English-Russian bilinguals align with baseline Russian speakers in their prominence ratings?

The second objective of the present study is to empirically assess the concurrent use of constituent order and tonal correlates of the nuclear pitch prominence during focus assignment in Russian. This is achieved by testing focus assignment preferences in the reference group of Russian monolinguals and the test group of English-Russian bilinguals.

To this end, in Experiment 2, we carry out a backward focus assignment task (originally implemented with English NSs in Breen et al., 2010). In this task, listeners use prosodic expression and constituent order in spoken test sentences as heuristics for detecting which word presents the most likely non-contrastive focus exponent in the given sentence. The following research questions are addressed: (3) Which cues do listeners rely on during focus assignment? (4) Do English-Russian bilinguals align with baseline Russian speakers in their use of prosodic expression and constituent order when assigning focus at phrasal level?

### 4.1 Experiment 1: the prominence identification task

Experiment 1 tested perception of phrasal prominence in simple transitive sentences presented without supporting context. Given that English-Russian bilinguals have previously demonstrated indeterminacy in auditory prominence identification (Luchkina et al., in press) and non-target-like, limited acceptance of focus configurations under the OVS constituent order (Ionin et al., 2023a), we compare word-level prominence ratings from monolingual speakers of Russian and English, recruited as reference raters, to those from a group of English-Russian bilingual listeners. Critically, the present study purposefully zooms in on the contribution of the prosodic expression, with a special focus on the tonal cues (per prior account of the Russian intonation by Bryzgunova, 1980), to perceived prosodic prominence at word level. For that reason, the stimuli sentences in Exp. 1 are presented for prosodic prominence identification as stand-alone, no context provided, utterance-long segments.

#### 4.1.1 Materials

The Russian stimuli sentences comprised 24 pre-recorded SVO sentences (e.g., 1b. repeated as 5a. below) and 24 pre-recorded OVS sentences (e.g., 2b. repeated as 5b. below) from Ionin et al. (2023a) and Luchkina et al. (in press). The nuclear pitch prominence in the pre-recorded test sentences (HL*) was invariably phrase-final, i.e., aligned with the object nominal in the SVO order and subject nominal in the OVS order. All subjects and objects were animate nouns. All objects contained an overt Accusative case marker. For the English version of the task, the Russian SVO test sentences (*n* = 24) were translated into English and audio recorded (see example 3 repeated in 6. below). 
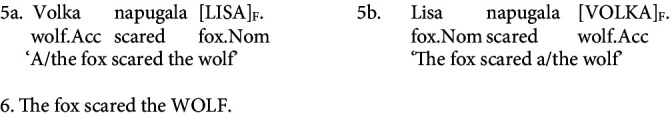


Both Russian and English item lists included fillers originally recorded and tested in Ionin et al. (2023b). Each filler was one sentence long and contained a contrastively accented word in variable phrasal positions (LH* in Russian; L + H* in English). In the Russian version of the task, fillers featured variable constituent orders. These included SV[O]_F_ and S[O]_F_V, [S]_F_VO and S[V]_F_O configurations (see examples 7a–d). English fillers were SVO sentences with variable placement of the nuclear pitch accent: [S]_F_VO, S[V]_F_O, SV[O]_F_. 
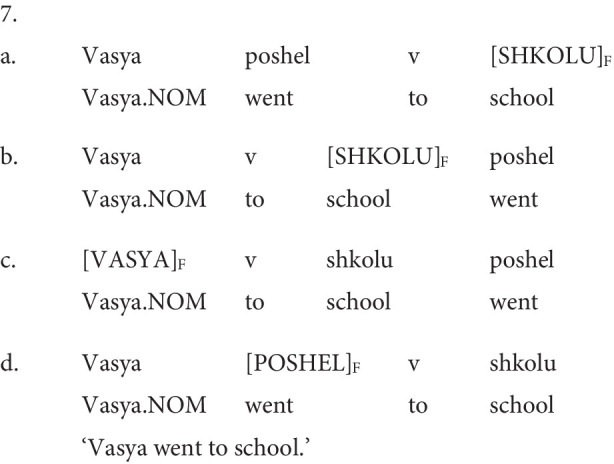


During stimuli recording sessions, on the speakers’ reading sheet, each target sentence was preceded by one-question-long context (see examples 1–4) which set the sentence-final noun in focus. The model speakers were instructed to read the question-answer pairs with natural intonation, with main prominence on the sentence-final noun. Only the answer component of each item (target or filler) was utilized in the listening tasks reported in this study.

The model speakers were female native speakers of Russian and English who did not participate in any of the tasks. The English speaker was not informed about the purpose of this study and was not linguistically trained. The Russian speaker served as an investigator on an earlier project involving the same set of stimuli (Ionin et al., 2023a) and was a graduate student in Linguistics when recordings were made.

The model speakers read the target sentences with neutral intonation, with main prominence on the sentence-final noun. For filler items, recorded subsequently, the location of the main phrasal prominence was indicated using UPPER CASE letters on the speaker’s reading sheet. Recordings were completed in a soundproof booth, at the University of Illinois Phonetics lab.

The 24 English target sentences were presented in a single item list, intermixed with 24 English filler sentences. There were two item lists in the Russian task each containing twenty-four fillers and 24 target sentences, 12 SVOs and 12 OVSs.

##### 4.1.1.1 Acoustic-prosodic analyses of the recorded stimuli sentences

The recorded audio was digitized at a sampling rate of 44 k, and manually annotated in Praat (Boersma and Weenink, 2024). Several tonal correlates of the main phrasal prominence were examined, including word-level pitch minima, maxima (Hz, st), and excursion size^5^ (st), extracted from each nominal constituent (subjects and objects).

All measures of interest were sampled twice. The first set of measurements was extracted from the stressed vowel in each content word in the experimental sentences. The second set comprised word-level measurements, which were not limited to the tonic vowel. For the inferential analyses in the present study, we opted to use word-level measures in set 2. This decision was based on earlier work on Russian, which found that the post-tonic syllable often aligns with a pitch peak or another critical element of the pitch contour [see Jasinskaja (2016), for further discussion].

In the Russian stimuli sentences (see Table 1), object pitch maxima exceeded those of subjects in the object-first OVS order but not in the subject-first SVO order. Similarly, pitch excursion over the objects was greater than those over the subjects in the OVS order, but not in the SVO order.^6^ While none of the examined acoustic-prosodic parameters conclusively demonstrated quantifiable evidence of prosodic augmentation in the vicinity of the phrase-final nuclear pitch-accented noun,^7^ visual inspection of the pitch contours over the sentence-final noun revealed consistency with the HL* intonational contour, in line with the analysis of the Russian intonation by Bryzgunova (1980), Zybatow and Mehlhorn (2000), see Šimík (in press) for more extensive discussion.

**Table 1:** Summary statistics of model speaker’s production data by constituent order and prosodic parameter; the Russian stimuli sentences.

Constituent order	measure	mean, Hz (st)	SD, Hz (st)
OVS	max f0, object nominal	362.3 (61.8)	56.72 (14.4)
	max f0, subject nominal	308.14 (50.19)	71.8 (17.44)
SVO	max f0, object nominal	307.47 (53.2)	63.9 (11.05)
	max f0, subject nominal	396.36 (68.5)	77.77 (13.5)
OVS	min f0, object nominal	191.01 (32.1)	9.45 (5.8)
	min f0, subject nominal	186.4 (30.4)	10.56 (7.83)
SVO	min f0, object nominal	182.2 (31.5)	9.11 (1.58)
	min f0, subject nominal	202 35.1	16.1 (2.8)
OVS	pitch excursion, object nominal (st)	10.88	2.98
	pitch excursion, subject nominal (st)	8.2	3.0
SVO	pitch excursion (st), object nominal	8.78	2.73
	pitch excursion (st), subject nominal	11.4	3.16

The acoustic-prosodic measures extracted from the English stimuli (see Table 2) paralleled those reported for Russian, but also included an additional set of measures sampled from the sentence-medial verbal constituent. This was deemed necessary because in the English version of the prominence identification task, the verb was often rated as prosodically prominent.

**Table 2:** Summary statistics of model speaker’s production data by constituent order and prosodic parameter; the English stimuli sentences^1^.

Constituent order	Measure	Mean, Hz (st)	SD, Hz (st)
SVO	Max f0 (Hz), Object nominal	294.02 (50.9)	161.68 (27.85)
	Max f0 (Hz), Subject nominal	365.34 (63.25)	117.29 (20.3)
	Max f0 (Hz), Verb	417.63 (72.3)	168.67 (29.2)
	Min f0 (Hz), Object nominal	97.67 (16.1)	15.92 (2.76)
	Min f0 (Hz), Subject nominal	192.18 (33.27)	18.89 (3.27)
	Min f0 (Hz), Verb	184.3 (31.91)	40.06 (6.94)
	f0 excursion (st), Object nominal	17.082	8.44
	f0 excursion (st), Subject nominal	9.7	3.82
	f0 excursion (st), Verb	13.03	8.05

^1A referee points out greater distribution in the tonal values in the recorded English stimuli, in comparison to the Russian stimuli sentences. The difference, in perceptual terms, indicates a livelier reading style of the English model speaker. The Russian speaker’s narrow pitch range and overall smaller pitch excursions represent the speaker’s understanding of “neutral intonation”.^

Analyses of the acoustic-prosodic expression in the English stimuli revealed that verbs had, on average, the highest pitch peak values [max f0 = 72st (SD =27.8st)], surpassing the highest pitch values over sentence subjects, on average, by 9st, and over objects, on average, by 21st. Meanwhile, sentence-final objects exhibited relatively lower pitch minima and maxima but displayed the greatest pitch excursion [mean = 17.1 (SD = 8.4 st)]. Where possible, visually inspected pitch contours over the sentence-final noun were consistent with the H* intonational contour, supporting prior analyses of English intonation (Katz and Selkirk, 2011).

**Figure 2:**
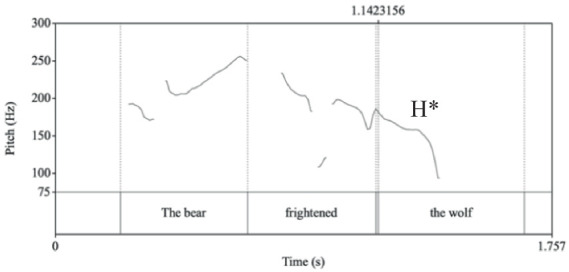
Illustrative pitch track of an English stimulus sentence (present study) containing a H* nuclear pitch accent.

Visual examination of the nuclear pitch peaks was not possible in all English stimuli sentences due to a high incidence of vocal fry in the vicinity of the phrase-final nuclear pitch-accented noun. A similar incidence of vocal fry in recorded English sentence stimuli has been reported by Yeung et al. (2019). Following Wolk et al. (2012), Yeung et al. (2019) discuss the intonational pattern, whereby the utterance-final nuclear accented noun exhibits pitch declination in combination with significant vocal fry, typical for expressing new information focus by young speakers of American English. The analysis of the English stimuli, recorded by a young female speaker of American English, aligns with this observation, despite the fact that our English model speaker did exhibit the more conventional H* contour, as depicted in Figure 2. We infer that in the English stimuli, the presence of vocal fry, coupled with pitch lowering, accounts for the relatively lower pitch peak values in the nuclear-accented nouns.

In summary, the analysis of model speakers’ performance data revealed a distinctive tonal quality in nuclear pitch-accented words, primarily attributable to pitch contours, rather than peak height or excursion height. This distinction was observed when comparing tonal measures of the nuclear-accented sentence-final noun with those of the sentence-initial noun. Notably, none of the investigated tonal parameters provided conclusive evidence of prosodic enhancement in the vicinity of the phrase-final nuclear pitch-accented noun.

These findings suggest that perceptual outcomes in the prominence identification task are likely to vary. Some listeners may expect to locate the nuclear-accented word phrase-finally, while others may seek a recognizable pitch contour or acoustic-prosodic expression at the word level to identify the main phrasal prominence. These variable expectations are reflected in the testable predictions outlined for Experiment 1 below.

#### 4.1.2 Participants

Data were obtained from three groups of linguistically naïve speakers, including baseline participant groups of Russian-speaking monolinguals (*n* = 29, mean age = 20.4) and English-speaking monolinguals (*n* = 68, mean age: 20.8). The monolingual speakers were recruited from among college student populations in Russia and in the US, respectively, and participated for course credit.

The third participant group included 29 English-Russian bilinguals (mean age = 36). The average age of exposure to English was 2.0 y.o; the age of exposure to Russian ranged between 0 and 30. All participants resided in the US, Canada, or Great Britain at the time of testing and declared English to be their native language as well as their preferred language for daily communication. Fifteen participants reported limited exposure to Russian via one or both parents. One participant reported that Russian was their native language, whereas English was their primary language. Thirteen participants reported completion of at least 2 semesters of formal classroom instruction in Russian as a foreign or heritage language. Seven additional bilingual participants were tested but eventually excluded due to extensive residence in a Russian speaking country (1 participant), failure to understand the task instructions (1 participant) and failure to meet the minimum proficiency requirements (5 participants). The English-Russian bilinguals were paid for their participation.

#### 4.1.3 Target language proficiency measures

The Russian language proficiency requirements for participant inclusion were established to guarantee comparable individual performance and avoid outcomes influenced by a deficit in TL proficiency, as previously noted by Luchkina et al. (in press) and in studies investigating focus perception in other languages (refer to Hoot, 2017 for relevant discussion). Furthermore, because the Russian stimuli involved a non-canonical constituent order, meaningful results can only be assured if the English-Russian bilinguals demonstrate above chance accuracy in interpreting the OVS stimuli as object-initial and subject-final, and not vice versa.

Bilingual speakers’ TL proficiency was evaluated using two independent measures, a 10-item test of morphological case previously implemented in Ionin and Luchkina (2019), Ionin et al. (2023a) and a 57-item cloze deletion test (Luchkina et al., 2021). The correlation between these two proficiency measures in the present study (Pearson’s *r*) reached 0.69 (*p* < 0.0001). The case check test assessed participants’ accuracy in discriminating between the nominative and accusative cases based solely on overt morphological markers. The cutoff score for the case test was set at 0.6, and for the cloze deletion test - at 0.7. The mean accuracy achieved on the case check was 0.87. (range: 0.6–1. SD = 0.16); the mean accuracy achieved on the cloze deletion test was 0.77 (range: 0.71–0.96, SD = 0.15).

#### 4.1.4 Procedure

All participants provided a written consent to participate and completed a language background questionnaire.

Participants were instructed to attentively listen to each target sentence and pay close attention to the prosodic expression in the model speaker’s read performance. Subsequently, participants were asked to select the word, in each target sentence, which they perceived as the most prominent, by clicking on it within the written sentence presented along with the audio recording.^8^

In the prominence identification task, each content word could be selected as prominent, for each test sentence. Prominence, in this context, was defined as a word-level attribute that directs the respondent’s attention more toward the prominent word compared to other words within the same segment. Instructions were presented in the dominant language of the task participants. Drawing on Cole et al. (2019, p. 120), in the English version of the task, prosodic prominence was characterized “as a word-level property leading certain words to have increased loudness, duration, pitch extremity, and ‘crisper’ articulation than the surrounding words.” The Russian monolinguals were provided the following adaptation of the Cole et al.’s (2019) definition of prominence: *“[…] select the word which the speaker highlighted by means of intonation. Such words are usually pronounced louder, longer, and with special voice timbre and may be regarded as key words in an utterance or phrase.”* Participants viewed two example items and completed three practice items, with feedback, before beginning the task. Participants completed this and the following tasks using Qualtrics online data collection platform.

#### 4.1.5 Testable predictions

We predicted that all participants would opt for the word with the most prominent prosodic expression, attributed to pitch accenting or another salient prosodic property. Given the nuanced nature of nuclear pitch prominence in the phrase-final position (e.g., Katz and Selkirk, 2011), this might result in varying perceptual preferences. For instance, a non-phrase-final word could be perceived as prominent and not the nuclear pitch accented nominal. This potential outcome would be substantiated by relatively higher values of the tonal measures extracted from non-phrase-final constituents in the test sentences, as discussed in 4.1.1.1.

We anticipated that participant performance may differ based on the dominant language. Specifically, the Russian monolinguals were predicted to demonstrate sensitivity to the acoustic-prosodic expression, at word level, in the recorded test sentences. Furthermore, because two types of nominal constituents, subjects and objects, aligned with the phrase-final, nuclear pitch-accented position, we also predicted that listeners’ judgements may be affected by constituent order in the test sentences as previously shown in Luchkina et al. (2015) and Luchkina and Cole (2021) who demonstrated that *ex-situ* words had a greater likelihood of being perceived as prominent by native Russian listeners. This suggests, for the OVS test sentences, a possibility for not just the nuclear accented subject, but also the fronted object, to be perceived as audibly prominent.

The English-Russian bilinguals were predicted to demonstrate sensitivity to the acoustic-prosodic expression at word level, due to transfer from the dominant language, more than to the constituent order when selecting the prominent word. This expectation arose from the lack of the OVS order in English.

#### 4.1.6 Results

We begin by reporting participant rates of perceived nuclear prominence in fillers, as an overall gauge of participants’ attention during the prominence identification task. As stated above, the filler items (see example 4) each featured an instance of narrow contrastive focus which occurred in various positions within a sentence (initially, medially, and finally). Across participant groups, the mean rates of perceived nuclear prominence in relation to contrastive focus ranged between 0.87 and 0.91. In the Russian version of the task, Russian NSs chose the nuclear-accented word as prominent in 88% of the items, and English-Russian bilinguals – in 0.91%. In the English version of the task, English NSs chose the nuclear accented noun as prominent in 87% of the fillers. The obtained rates of perceived nuclear prominence, consistently high independent of the language of the task or the participant group, serve as evidence of on target, accurate performance by all participants.

Next, we examine the rate of perceived nuclear prominence in the test sentences each containing an instance of non-contrastive focus. We proceed by first reporting results obtained from the two groups of monolinguals (reference) speakers, and next - from the group of English-Russian bilinguals (see Table 3) for results summary.

**Table 3:** The mean rate of perceived nuclear prominence (means, SD) in the tested groups.

Prominent word category:	Sentence-final noun (nuclear accented)	Sentence-final noun by constituent order		Verb	Sentence-initial noun
English monolinguals	0.08 (0.27)			0.2 (0.4)	0.73 (0.45)
Russian monolinguals	0.35 (0.5)	SVO: 0.4 (0.5)	OVS: 0.3 (0.46)	<0.02	0.64 (0.4)
English-Russian bilinguals	0.45 (0.5)	SVO: 0.46 (0.5)	OVS: 0.43 (0.5)	<0.03	0.52 (0.5)

In a test sentence “The wolf scared the fox,” sentence-final noun” refers to “fox,” ‘sentence-initial noun’ refers to “wolf,” and “verb” refers to “scared.” “Sentence-final noun by constituent order” refers to objects in SVO and subjects – in OVS Russian stimuli sentences.

The dependent measure in the data analyses for Experiment 1 is the likelihood of the nuclear pitch accented noun being rated prominent by the listeners. Inferential analyses modeling the likelihood of perceived nuclear prominence in the test sentences consisted of multinomial mixed-effects logistic regressions with *constituent order* (Russian data only), *cloze test score* (bilinguals’ data only), and tonal measures of *pitch maxima, minima,* and *pitch excursion* entered as fixed effects. All tonal measures were coded separately for subjects and objects. The random effects for each model consisted of *participant* and *test item* (slopes and intercepts).

##### 4.1.6.1 The English monolinguals

In the data obtained from the English monolinguals, the mean rate of perceived object prominence reached 0.08 (SD = 0.27) revealing an overwhelming preference to select the sentence-initial subject as prominent (mean = 0.73, SD = 0.45). Additionally, the verb was identified as prominent in approximately 20% of the test sentences (mean = 0.19, SD = 0.4). A mixed-effects multinomial logistic regression further revealed that, relative to the baseline category of the clause-final nuclear accented object, both the clause-initial subject and the clause-medial verb in the English stimuli were more likely to achieve perceived prominence, based on their acoustic-prosodic expression.

Among the tested acoustic-prosodic parameters, including the tonal measures, higher values of pitch minima over subjects and verbs (*z*_subjects_ = −5.58, *z*_verbs_ = −4.63, respectively, all *p*-values <0.0001), as well as greater pitch excursion over these constituent categories (*z*_subjects_ = −5.0, *z*_verbs_ = −3.66, respectively, all *p* values <0.0001), were negatively predictive of the nuclear accented object prominence, coded as base outcome in the regression model.

##### 4.1.6.2 The Russian monolinguals

Here, we examine the rate at which object nominals in the SVO test sentences and sentence-final, nuclear-pitch accented subjects in the OVS test sentences, were identified as prominent by monolingual Russian listeners. Because of a very low incidence of perceived verb prominence (<2% of all ratings) in the Russian monolinguals’ data, the present discussion takes into account subject and object nominals only.

The mean rate of perceived nuclear prominence was 0.4 (SD = 0.5) in the SVO stimuli and 0.3 (SD = 0.46) in the OVS stimuli (overall task mean rate of nuclear prominence = 0.35, SD = 0.5). A mixed-effects logistic regression assessed the contribution of the acoustic-prosodic expression in subject and object nominals to their respective prominence rates. The fixed effects of interest included constituent order and the tonal measures of pitch. In this analysis, local pitch minima were excluded due to a collinearity effect. The pitch measures from subject and object nominals, which could be phrase-initial or phrase-final due to the constituent order manipulation, were coded separately and further interacted with constituent order.

The rate of perceived nuclear prominence was greater in the SVO sentences (*z* = 2.14, *p* = 0.03). In the SVO order, higher pitch maxima and excursion in sentence-final nominals were positively correlated with the probability of nuclear prominence (pitch maxima: *z* = 1.99, *p* = 0.05; pitch excursion: *z* = 2.19, *p* = 0.03).

##### 4.1.6.3 The English-Russian bilinguals

This next set of results in the present experiment pertains to the performance of the English-Russian bilinguals. Because of a low incidence of perceived verb prominence (<3% of ratings) in the English-Russian bilinguals’ data, the present discussion takes into an account subject and object nominals only.

The mean rate of perceived nuclear prominence reached 0.45 (SD = 0.5). Differences in the mean rate of nuclear prominence due to constituent order appeared numerically low: SVO = 0.46 (SD = 0.5); OVS = 0.43 (SD = 0.5). A mixed-effects logistic regression evaluated the contribution of the acoustic-prosodic expression in subject and object nominals to their respective perceived prominence rate. Once again, local pitch minima were excluded due to collinearity. The model tested an additional main effect of participants’ TL proficiency, as measured by means of a cloze test which all bilinguals speakers completed as a part of the present study.

The analysis returned no effect of constituent order. Both tested pitch measures (maxima and excursion size) yielded significant main effects (omitted for brevity) and interacted with constituent order, as follows. In the SVO order, the size of pitch excursion (st) as well as pitch peak height over the sentence-final object were positively associated with the likelihood of the nuclear pitch prominence (excursion: *z* = 4.72; *p* < 0.0001; peak height: *z* = 4.24. *p* < 0.0001). The size of pitch excursion and peak height over the sentence-initial subject, on the contrary, were negatively associated with the likelihood of perceived nuclear pitch prominence (pitch excursion: *z* = −-4.14, *p* < 0.000; pitch maxima: *z* = −2.15, *p* < 0.03;). Participants’ performance on the multiple-choice cloze deletion score was positively, albeit weakly, predictive of how likely they were to select the nuclear accented noun as prominent, across the tested constituent orders (*z* = 1.82, *p* = 0.07).

##### 4.1.6.4 Cumulative analysis of experiment 1 data

One final component of the present analysis is the model fit to the data obtained from all participants who completed the Russian version of the task, including the Russian monolinguals and the English-Russian bilinguals. The joint analysis revealed no main effect of language background or constituent order but highlighted the significant contribution of the tonal measures of nuclear prominence to perceived prominence ratings. The pitch peak height over sentence-initial subjects was negatively predictive of perceived nuclear prominence (*z* = −4.42, *p* < 0.0001), while higher pitch maxima over the sentence-final objects were positively associated with the likelihood of perceived nuclear prominence (*z* = 5.04, *p* < 0.0001). Similarly, all Russian-speaking participants were sensitive to the size of the pitch excursion over sentence-initial subjects (*z* = −4.35, *p* < 0.0001) and sentence-final objects (*z* = 5.95, *p* < 0.0001).

#### 4.1.7 Discussion

Experiment 1 pursued the following questions: (1) What cues underly percept of prosodic prominence in simple transitive Russian sentences? (2) Do English-Russian bilinguals pattern with baseline Russian speakers in their prominence ratings?

In order to answer these questions, listeners provided ratings of perceived word-level prominence in the experimental stimuli based on acoustic-prosodic expression alone, i.e., in the absence of context cues. The experimental sentences were designed with the nuclear pitch accent on the sentence-final word, aligning with the preferred location of the main phrasal prominence in Russian and English.

We predicted that all participants would demonstrate sensitivity to tonal measures contributing to pitch movement at the phrasal level in both English and Russian. Asymmetric rates of nuclear pitch prominence in SVO vs. OVS order were anticipated for the Russian monolinguals but not for the English-Russian bilinguals. This difference in prediction arises from the distinct grammars underlying each language: In Russian, phrasal prosody interfaces with constituent order in expressing information structure, leading constituent order to contribute to perceived word-level prominence [see Luchkina and Cole (2021) for a recent empirical investigation]. In contrast, in English, prosodic cues serve as the primary means of signaling prominence, while constituent order flexibility remains highly limited.

As predicted, all participants exhibited sensitivity to tonal measures in the modal speakers’ read performance, including local pitch minima and maxima and the pitch excursion at word level. These cues supported near-ceiling rates of perceived nuclear prominence in filler sentences each featuring a contrastively accented word. These high rates of nuclear prominence in relation to the contrastive accenting patterns (LH* in Russian, L + H* in English) are consistent with recent research addressing contrastive focus in English (Bishop, 2012) and in Russian (Ionin et al., 2023b). The latter study, specifically, examined perceived contrastive focus prominence and identification in L1 and L2 Russian. Results reported by Ionin et al. (2023b) support that both Russian monolinguals and Russian-English bilinguals successfully identify contrastive foci in read recorded speech as prominent.

While this study presented fillers without supporting context, we attribute the high rates of perceived nuclear prominence in the filler sentences to the prosodic characteristics of the contrastively accented word. As reported in Bryzgunova (1980), contrastive focus in Russian receives a distinct prosodic contour, referred to as the non-neutral IK-2. Under the contour in question, the nuclear prominence may occur anywhere in the utterance, non-nuclear accents as well as pitch downstep tend to be eliminated, and the accented syllable is produced with particularly high intensity.

In a similar vein, Bishop (2012) argues for considerably greater prosodic prominence of contrastive focus (in comparison to non-contrastive focus) in English. An investigation by Cole et al. (2019) lends empirical support for this view. Cole & colleagues conducted a prominence rating task using recorded excerpts of connected English speech. The rate of perceived nuclear prominence (downstepped H*) in neutral intonation sentences in Cole et al.’s study reached approximately 0.3 and further reached approximately 0.5 in the sentences featuring an instance of narrow contrastive focus (L + H*).

The relatively higher prominence rates obtained by Cole et al. and in the present study may be attributed to the fact that in the former study, listeners were presented with stretches of connected discourse, whereas in the present study – utterance-long segments presented without context.

While acoustic-prosodic predictors continued to play a determinant role during prominence identification in the test items recorded with neutral intonation, most listeners were unlikely to select the nuclear accented noun as prosodically prominent. More specifically, the English monolinguals identified the phrase-initial subject nominal as prominent in 72% of the stimuli sentences and rated the verb as prominent in 20% of the test items.

The relatively high rate of perceived subject prominence in the English stimuli sentences could be attributed to several other factors. Branigan et al. (2008) make a compelling argument for the special perceptual status of sentence subjects in English, due to their agentive role and animacy. Even though all subjects and objects in the experimental sentences were animate nouns, in the absence of context, some listeners possibly treated the stimuli sentences as instances of broad focus (i.e., all new information). As the information status remained constant across each test sentence, the grammatical function, in line with Branigan’s proposal, could have further contributed to a prominent reading of the subject nominals.

At the same time, systematically reduced tonal measures in the vicinity of the phrase-final objects in the English stimuli have led to their relatively lower perceived prominence ratings. This proposal is further supported by the fact that (1) listeners were explicitly instructed to respond to the relative prosodic prominence at the word level during the prominence identification task and (2) phrase-finally, the tonal expression of pitch prominence is naturally acoustically reduced [see Katz and Selkirk (2011) and Yeung et al. (2019) for more discussion].

The relatively high rate of perceived subject prominence in the English stimuli sentences could be further attributed to several other factors. Branigan et al. (2008) make a compelling argument for the special perceptual status of sentence subjects in English, due to their agentive role and animacy. Even though all subjects and objects in the experimental sentences were animate nouns, in the absence of context, some listeners possibly treated the stimuli sentences as instances of broad focus (i.e., all new information). As the information status remained constant across each test sentence, the grammatical function, in line with Branigan’s proposal, could have further contributed to a prominent reading of the subject nominals.

Results from the monolingual English speakers overlapped with those obtained from the Russian monolinguals in several ways. Specifically, both groups were responsive to the tonal measures in the model speakers’ read performance and prioritized subject prominence over object prominence. Specifically, pitch excursion size predicted the likelihood of subject nominal prominence in both listener groups. Likewise, all monolinguals responded to the relative height of the pitch peaks over the phrase-final nominal constituents (as revealed in the joint analysis of the Russian task data) when selecting the prominent word.

The native-like perception of the tonal correlates of prominence in Russian may be attributed to positive transfer from the dominant language and, for some bilingual participants, to early exposure to Russian deemed critical for integrating phrasal prosody with the rest of the utterance, during listening (Laleko, 2024).

The impact of constituent order is where the performance of the two Russian speaking groups appeared to diverge. Specifically, the rate of perceived nuclear prominence in the Russian monolinguals’ data was consistently higher, by 10% on average, in the SVO stimuli sentences compared to the OVS sentences. This difference stemmed from the tendency by the Russian monolinguals to select the sentence-initial object as prominent in the non-canonical OVS order. This result is consistent with the perception and production of alternate constituent orders in Russian previously investigated by Luchkina and Cole (2016, 2021). Luchkina and Cole (2016) reported evidence of comprehensive prosodic augmentation by Russian native speakers of words occurring *ex-situ*, such as the sentence-initial object in the OVS sentences tested in the present study [see Vainio and Järvikivi (2006), Patil et al. (2008), and Luchkina et al. (2015) for similar findings in other flexible word order languages]. A follow up investigation by Luchkina and Cole (2021) found that *ex-situ* words in Russian are also more likely to be perceived as prominent during listening comprehension by adult native listeners.

In contrast to the results obtained from the monolingual speakers, the perception data from English-Russian bilinguals revealed a null effect of constituent order, despite satisfactory accuracy on the case check task and the cloze deletion test. Considering that both English and Russian utilize nuclear pitch prominence for marking focus, alternations in constituent order present a crucial asymmetry between the two languages and serve as a central axis for drawing a comparison between the speaker groups under investigation. We revisit the effect of constituent order on the rate of perceived nuclear prominence in the general discussion section 4.

### 4.2 Experiment 2: backward focus assignment

In Experiment 2, we tested if English-dominant Russian bilinguals can integrate nuclear pitch prominence and constituent order with discourse context to determine the word in sentence focus. The task is modeled after Breen et al. (2010) who previously investigated focus assignment in English, with functionally monolingual native English speakers.

#### 4.2.1 Materials

Russian native speakers and proficient English-Russian bilinguals were compared on the use of the tonal focus correlates and linear ordering of sentence constituents during focus assignment.

Materials consisted of the 24 sentences tested in experiment 1 [see (8)], and 48 wh-questions [see (8.1) and (8.2)]. Each test sentence was paired with two wh-questions which cued the focal status of the subject or the object nominal in the test sentence. To illustrate, in the example (8) below, the object question in (8.1) correctly sets the nuclear pitch accented object ‘VOLKA’ (eng.: “wolf.GEN”) in focus; conversely, the subject question in (8.2) incorrectly sets the subject noun ‘LISA’ (eng.: “fox.NOM”) in focus. 
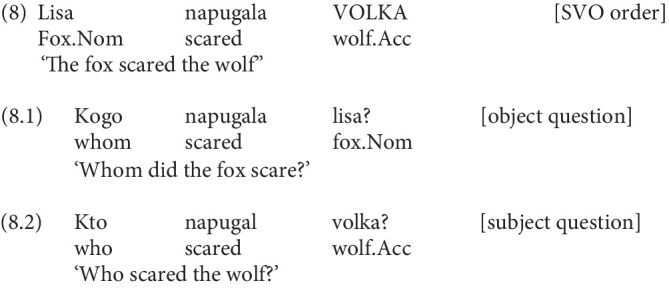


The filler sentences from experiment 1 were paired with two wh- or yes-no questions, in the same format as the test items.

#### 4.2.2 Participants

The Russian-speaking participants who completed the auditory prominence identification task continued to experiment 2, including the Russian monolinguals (the reference group) and the English-Russian bilinguals (the test group).

#### 4.2.3 Procedure

In each trial, listeners had to decide whether each test sentence was a response to an object or a subject question to determine which of the two nouns, the subject or the object, the speaker intended as the sentence focus. They were instructed to select a context question which best matched the target sentence, using the two provided options. One of the options set the nuclear accented word in the target sentence in sentence focus (Match), while the other– assumed a focal reading for a non-nuclear accented noun (Mismatch).

The experimental sentence was presented auditorily and the 2 context questions were presented side by side, in writing. Participants listened to the target sentence and selected the matching question with a mouse click.

#### 4.2.4 Testable predictions

In the backward focus assignment task, we investigate the rate at which new information foci, nuclear pitch accented in the sentence-final position, were successfully disambiguated by listeners, as indicated by the rate of choosing matching contexts over non-matching ones. The most salient cues to sentence focus made available in the sentence stimuli included constituent order and tonal correlates in phrase-final subject and object nominals.

Because information structure serves as an interpretative license for constituent re-ordering in Russian, we predicted that the Russian monolinguals would exhibit preference to assign focus to the nuclear-accented noun sentence-finally, across the tested constituent orders. This same prediction can be further extended to the bilinguals’ group if bilinguals at higher TL proficiency successfully associate word order with distinctions in the information structure. If, on the contrary, an effect of constituent order emerges in the bilinguals’ data, it would be indicative of transfer from the dominant grammar, where the said effects of information structure on constituent order are not found.

Despite the lower rates of perceived phrase-final prominence obtained during nuclear prominence identification, we nevertheless anticipated above-chance rates of matching context-answer pairings due to the qualitatively different nature of the task at hand. While not instructed to attend closely to the intonation in the target sentences, participants were expected to perceive the stimuli more holistically and take information structure in the question-answer pairs into an account. Critically, this expectation holds both for English-dominant and Russian monolingual speakers, owing to an overlap in (1) the default, phrase-final placement of the nuclear pitch prominence in both these languages and (2) the tonal correlates of the nuclear pitch prominence marking non-contrastive focus in Russian and English.

#### 4.2.5 Results

As with Experiment 1, we first report the rate of focus assignment in the filler items (see example 4) which featured an instance of narrow contrastive focus occurring in various positions within a sentence (initially, medially, and finally). Native Russian speakers’ target focus assignment rate reached an average of 0.77 and varied among the different types of fillers (range: 0.70–0.83). English-Russian bilinguals demonstrated comparable performance, at the average rate of on target contrastive focus assignment of 0.82. These results support that all participants paid attention and understood the task instructions.

**Figure 3:**
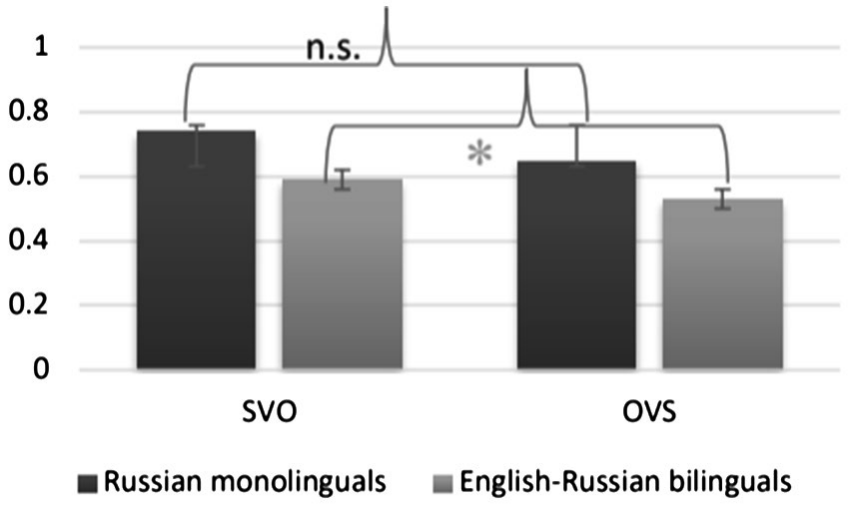
Mean accuracy rate (*y*-axis) obtained in the backward focus assignment task in the Russian stimuli sentences. Error bars represent Standard Deviation.

The overall rate of on target focus assignment in non-contrastive focus items reached 0.63 (SD = 0.48). The Russian monolinguals achieved the mean accuracy of 69.4 (SD = 0.46). As shown in Figure 3, the mean accuracy rate was numerically higher on SV[O]_F_ items (mean = 0.74, SD = 0.44) than on OV[S]_F_ items (mean = 0.65, SD = 0.48). The English-dominant bilinguals were 0.56 accurate overall (SD = 0.5). Their accuracy also differed across the tested constituent orders: SVO: 0.59 (SD = 0.49); OVS: 0.53 (SD = 0.5).

The constituent order fixed effect in the monolingual speakers’ data did not reach significance when evaluated in a mixed-effects logistic regression. The same analysis additionally assessed the contribution of the tonal focus correlates to the likelihood of accurate backward focus assignment, as well as their interaction with constituent order. The tonal measures of pitch maxima and excursion size in object nominals were positively predictive of accurate focus assignment to the nuclear pitch accented word under the SVO constituent order (object pitch excursion: *z* = 2.27, *p* = 0.02; object pitch maxima: *z* = 1.87, *p* = 0.06) by the Russian monolingual speakers.

**Figure 4:**
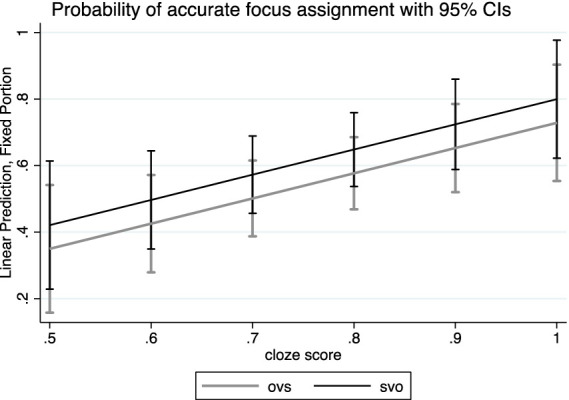
Likelihood (linear prediction) of accurate focus assignment in bilingual speakers’ data. Top line: SVO stimuli sentences; lower line: OVS stimuli sentences. Error bars represent Standard Deviation.

An analogous model fit to the English-Russian bilinguals’ data revealed significant main effects of constituent order (*z* = 1.97, *p* = 0.05), as displayed in Figure 4 and cloze test score (*z* = 2.5, *p* = 0.01). Furthermore, pitch peak height over the subject nominals negatively predicted the accurate choice of the context question (*z* = −1.99, *p* = 0.05) in the SVO order.

The final component of Experiment 2 inferential analyses is a mixed effects model fit to the entirety of the Russian speakers’ data, with participants’ dominant language entered as a fixed effect. The model returned a significant main effect of participants’ dominant language (*z* = 2.22, *p = 0.*03), reflective of a more accurate performance of the Russian monolinguals on backward focus assignment. The tonal measures extracted from the object nominal interacted with constituent order (pitch excursion: *z* = 1.95, *p* = 0.05; pitch maxima: *z* = 2.16, *p* < 0.03;) and predicted accurate focus assignment to sentence-final objects in SV[O]_F_ test sentences. The size of pitch excursion over the sentence-initial subjects was negatively predictive of the likelihood of focus assignment to the nuclear-accented noun in SV[O]_F_ test sentences (*z* = −2.35, *p* = 0.02). Finally, the effect of constituent order approached significance (*z* = 1.8, *p* = 0.07), driven by the more robust effect obtained in the bilingual participants’ data but lacking in the data from the baseline speakers.

#### 4.2.6 Experiment 2: discussion

In Experiment 2, we examined whether English-Russian bilinguals demonstrate alignment with native Russian speakers in their use of prosodic features and constituent order when assigning focus at the phrasal level. To this end, participants completed a backward focus assignment task in which they were presented with target sentences alongside two context options. The position of the nuclear pitch-accented word in each target sentence was invariably sentence-final. Listeners were tasked with selecting the context question that would accurately place the sentence-final nominal in focus.

We predicted the Russian monolinguals to rely on the acoustic-prosodic cues, in combination with constituent order, when performing backward focus assignment. As long as both tested constituent orders conform to the same interpretative license, the listeners should anticipate sentence focus phrase-finally and in alignment with the default, phrase-final nuclear prominence lending site. This logic supports the expectation of comparable focus assignment accuracy across the tested constituent orders. This prediction, if borne out, goes against the observed effect of constituent order in the monolinguals’ prominence identification results obtained in Experiment 1. Furthermore, we expected English-Russian bilinguals to demonstrate performance above chance levels. This prediction is supported by satisfactory assessment outcomes of their proficiency in the TL, as well as the transfer of pitch marking for sentence focus from the dominant language.

Listeners demonstrated sensitivity to the tonal correlates of sentence focus, which systematically contributed to the choice of the matching context for both groups. Different listening patterns emerged, whereby the Russian monolinguals attended to the tonal properties of object nominals, such that greater pitch excursion cued object focus, irrespective of constituent order, and higher pitch peaks further supported object focus assignment under the baseline SVO order. In contrast, English-Russian bilinguals closely tracked the relative pitch prominence of the sentence-initial subject nominals in the SVO order, such that their pitch peak height was inversely associated with the likelihood of sentence-final object focus.

We interpret the listening pattern of English-Russian bilinguals to be influenced by transfer from their dominant language. In English, where constituent order flexibility is limited, it is more likely for the nuclear prominence to occur in variable phrasal positions, i.e., non-utterance-finally. This may have led our bilingual listeners to anticipate, on a probabilistic basis, a non-sentence-final nuclear pitch accent in the stimuli sentences. While this interpretation is tentative, it aligns with the higher rate of assigned subject foci in SVO order in the English-Russian bilinguals’ data (0.4), compared to the lower rate (0.26) – in the Russian monolinguals’ data. It’s noteworthy that Russian monolinguals, too, remained open to the possibility of subject focus in the SVO order but used pitch excursion (rather than peak height) as a leading tonal correlate supporting subject focus assignment to the sentence-initial subject. This unified analysis of the backward focus assignment data from both participant groups further supports the contribution of the tonal correlates of nuclear prominence in Russian, positively predicting accurate focus assignment across both tested constituent orders.

The performance of the two groups diverged when considering the effect of constituent order systematically varied in the stimuli sentences. All participants appeared more inclined to assign focus to the clause-final object nominal under the baseline SVO order. While the difference in the mean rates of accurate focus assignment in SVO vs. OVS stimuli sentences was numerically greater in the monolingual participants’ data, it did not reach significance.

The null effect of constituent order in the monolinguals’ data aligns with our prediction, indicating that the preferred strategy for monolingual listeners was to assign focus to the phrase-final nominal, which naturally aligns with the nuclear prominence lending site in Russian (Neeleman and Titov, 2009). This interpretation is critically supported by the fact that the Russian monolinguals exhibited a preference to assign focus to the sentence-final subject in the OVS stimuli sentences.

On the contrary, English-Russian bilinguals consistently showed a preference for assigning focus to the sentence-final object nominal in the SVO stimulus sentences. Simultaneously, they demonstrated a higher rate (47%) of assigning focus to the object in the OVS order. As expected, the bilinguals’ distinct approach to focus assignment was mirrored in their performance on the cloze deletion test, utilized as a gauge of target language proficiency. Specifically, bilinguals who performed well on the cloze test were more likely to assign focus to the nuclear accented word.

Adding further support to the qualitatively different approach to focus assignment in the two groups, a significant main effect of dominant language emerged in the unified analysis of the data, indicating an overall stronger tendency among the Russian monolinguals to assign focus to the sentence-final nominal constituent, irrespective of the constituent order. These findings support the prediction that Russian monolingual speakers relied on constituent order as a heuristic during focus assignment more than the English-dominant bilinguals.

## 5 General discussion

The present study investigates bilingual competence in the domain of information structure in Russian, a free word order, pitch-accenting language. The primary focus is on the simultaneous use of intonational prominence and constituent order as means of encoding sentence focus by English-Russian bilinguals with English as their primary or dominant language. The population of interest has previously demonstrated varied acceptability of non-SVO orders in Russian (Laleko, 2022; Ionin et al., 2023a) in conjunction with non-target-like perception of prosodic prominence used to mark non-contrastive narrow focus in object-final and subject-final transitive sentences (Laleko, 2024; Luchkina et al., in press).

The added complexity in relation to non-contrastive new information focus in Russian motivates the analysis of reference data from adult monolinguals commanding each of the languages of our bilingual participants. In the present study, this leads us to include monolingual Russian and English speakers whose auditory perception data and focus assignment data are used to establish baseline against which we then compare the results from the bilinguals.

The first listening task tested participants’ perception of the main phrasal prominence in a series of simple transitive sentences. Given the often-subtle nature of the acoustic-prosodic cues in the expression of a phrase-final nuclear pitch accent, we aimed to determine if listeners perceive the accented word as prominent based on its tonal expression. Additionally, we investigated whether the perception of phrasal prominence is influenced by the linear order of sentence constituents in Russian, in comparison to a fixed constituent order in English. Because a significant contribution of discourse context toward prominence identification has been previously reported by Luchkina et al. (in press), we chose to center the present investigation on the auditory perception of prominence in the absence of context cues.

The rate of perceived nuclear prominence was significantly lower (<10% of all ratings) in the data from monolingual English speakers who readily rated the sentence-initial nominal or the verb as more prosodically prominent than the sentence-final pitch-accented nominal. As far as the prosodic expression is concerned, the low rate of sentence-final nuclear prominence in the English stimuli sentences can be attributed to the frequent occurrence of vocal fry in the vicinity of the sentence-final object which often compromised the realization of the intended pitch contour and rendered the sentence-final object less prosodically prominent compared to the rest of the sentence.

In Russian, analyses of the tonal measures in the sentence stimuli revealed that the phrase-final noun, despite being nuclear pitch-accented, appeared less prosodically expressive compared to the non-phrase-final material. Nevertheless, in comparison to the English reference group, the Russian monolinguals were five times more likely to perceive the sentence-final, nuclear accented nominal as prominent in the baseline SVO stimuli sentences and nearly four times more likely—in the subject-final OVS order. Results from the English-Russian bilinguals did not align fully with either monolingual reference group. First, there was a very low incidence of verb prominence, unlike in the English monolinguals’ data. Second, bilinguals’ ratings were unaffected by constituent order in the test sentences, unlike in the Russian monolinguals’ data. Numerically, bilingual listeners were more likely to rate the nuclear-pitch accented noun as prominent, albeit the overall rate of perceived nuclear prominence remained under 50%.

The relatively lower rates of perceived nuclear prominence obtained in all participant groups appear even more notable considering that both Russian and English are known to default to phrase-final nuclear pitch prominence (Bryzgunova, 1980; Beckman and Pierrehumbert, 1986) and warrant further analysis of the individual contribution of tonal cues to nuclear pitch prominence in each listener group. At the same time, these results point to the probabilistic nature of perceived prosodic prominence during listening comprehension. The term “probabilistic” in the context of the prominence identification task administered in the present study translates into notable levels of individual variation in perception, stemming from distinct approaches to prominence identification adopted by linguistically naïve listeners. This variability suggests that some of the listeners tested in the present study prioritized prosodic expression as the primary “pathway” to prominence, while others relied on grammatical function, information status, and other discourse cues [see Branigan et al. (2008), Watson (2010), and Cole (2015) for further discussion]. Although all listener groups were explicitly instructed to focus on prosodic cues to determine prominence, some may have, in principle, evaluated perceived information prominence holistically, i.e., considered fundamentally non-prosodic cues to prominence, as discussed here and in section 3 above.

Our results align with a recent English study by Yeung et al. (2019) who established a largely probabilistic mapping between the cues used by L1 English speakers to express focus in elicited production and by listeners - during auditory comprehension of recorded speech. Similar findings on Russian were reported by Luchkina and Cole (2016) in an investigation of prosodic prominence correlates in read recorded speech by 15 native Russian speakers. The study found that several speakers failed to prosodically augment words which were referentially new in read discourse — a finding which parallels that of Yeung et al.’s (2019) study on English.

The probabilistic nature of perceived nuclear prominence in the present study may be further attributed to the fact that out stimuli sentences were presented without context against which the nuclear status of the pitch accent over the sentence-final noun could be interpretatively validated. As a result, listeners may have developed different heuristics leading to distinct prominence percepts. For example, some respondents may have been sensitive to phrasal prosody, which includes downstepping in pitch across an utterance. This feature supports greater perceptual prominence for words occurring earlier in the string, as opposed to the nuclear-accented, sentence-final word. This interpretation is supported by the high rate of perceived prominence associated with the sentence-initial noun reported by most listeners. Other participants may have relied on known information structural templates shared by Russian and English, since in both these languages, discourse-given information tends to be placed early in the utterance, while discourse-new information often appears at the end. This pattern supports a prominent reading of the utterance-final, nuclear-accented noun.

By considering these different heuristics, we can better understand the variability in listeners’ prominence percepts. Findings of Experiment 1 lead us to propose that a unity of prosodic cues and discourse heuristics is what may be necessary for a full-fledged percept of nuclear prominence to emerge within a listener. To test this proposition, one may require conducting an additional task asking the listeners to point out the most prominent word, as opposed to the most prosodically prominent word, while making the context available. A similar design has been previously implemented in Luchkina et al. (in press) where it gave rise to prosodic correlates and discourse cues to prominence being co-interpretable by listeners.

In Luchkina et al. (in press), Russian monolinguals and English-Russian bilinguals were tested using brief question-answer exchanges. The question sentences systematically set either the subject or the object in the SVO and OVS targets in sentence focus. The same target sentences were used as in the present study, giving rise to pragmatically felicitous and non-felicitous exchanges. In the non-felicitous items, the question sentence placed focus on the sentence-initial noun, which clashed with the sentence-final nuclear prominence in the answer sentence.

By manipulating context felicity, Luchkina et al. found that in felicitous question-answer pairings, the rate of nuclear prominence was greater, due to context unambiguously reinforcing the pitch accenting status of the sentence-final noun. The non-felicitous context, however, set a non-nuclear accented word in focus and thereby made listeners less likely to rate the nuclear-accented word as prominent. Despite the more variable proficiency levels of the English-Russian bilinguals tested by Luchkina et al. (in press), a robust effect of context felicity emerged, highlighting the tight interplay between context and prosodic cues in the perception of phrasal prominence. For instance, the rate of nuclear prominence in OVS targets dropped by 40% when a non-felicitous context was provided.

Since the present study investigates the role of intonational cues to prominence, discourse context was made unavailable. As a result, participants demonstrated greater reliance on prosodic expression.

Despite the overall lower incidence of perceived nuclear prominence reported in Experiment 1, all listener groups were responsive to word-level acoustic-prosodic tonal expression, which often rendered a non-phrase-final element prosodically distinct, even in the absence of a nuclear accent. More specifically, all listeners demonstrated sensitivity to local pitch maxima and a relative size of pitch excursion, independent of the language of the task. Numerically comparable rates of perceived nuclear pitch prominence obtained from the bilingual raters provide evidence of successful transfer in the domain of phrasal prosody from the dominant language (English), even though the tonal signatures of nuclear accents in the English (H*) and Russian (HL*) stimuli sentences were prosodically distinct.

We conclude that, phrase-finally, the nuclear pitch-accented status might not be as straightforward from a perceptual perspective, particularly in the absence of context cues that delineate the information structure of the utterance at hand and may further enhance the prominent status of the sentence-final word.

Bishop (2012) is an illustrative empirical study which further supports this proposal using data from English. In the prominence rating task administered by Bishop (2012), listeners were presented with spliced productions of SVO sentences in which the entire VP was in broad focus (both verb and object were accented) paired with contexts which only supported the focal status of the object. Following this manipulation, the listeners reported hearing the object as more prominent than the verb, and thereby revealed a robust effect of context cues and listener-based expectations of prosodic prominence in connected discourse.

We conclude that the construct of word-level prominence prominence extends beyond the specific focus of this study and is both conceptually and perceptually broader than the dependent measure examined in Experiment 1 (i.e., the rate of perceived nuclear prominence) and its critical predictors (tonal measures of nuclear accent and phrasal placement of the prominent word). Further investigations into the psychological, expectation-driven nature of perceived prominence coincidental with new information focus in various phrasal positions in Russian are warranted, given the probabilistic relationship between production-perception data reported in the present study and related work.

Experiment 2 asked listeners to identify the word in each test sentence that could plausibly serve as the focus exponent and provided listeners (albeit in a reverse fashion) with two contexts to choose from. By offering context alternatives, the the focus assignment task altered the participants’ listening experience and made them more likely to perceive nuclear prominence through the lens of the provided discourse scenarios. It is noteworthy that both tested groups responded to the task similarly, by accepting the focal reading of the nuclear-accented noun for the majority of the stimulus sentences (cf. Russian monolinguals: 70%; English-Russian bilinguals: 56%).

Of particular interest to the present study is how prosodic prominence identification and focus assignment proceed in the subject-final OVS order in Russian. Previously, Hoot (2017) reported lower acceptability of phrase-final representational subject foci (OV[S]_F_), in comparison to phrase-initial ([S]_F_VO), by native and heritage speakers of Mexican Spanish. Kallestinova (2007), however, reported an opposite pattern of relative acceptability in native Russian speakers who preferred the subject-final order when the subject was discourse-new. This preference may be accounted for by the fact that placing the subject nominal phrase-finally aligns it with the default location of the main phrasal prominence and, by doing so, effectively signals its focal status during listening.

An opposite pattern of acceptability judgments in Russian was recently discussed in Laleko (2024), where 14 baseline speakers equally accepted the prosodic encoding of sentence foci occurring *in situ*, as well as focus encoding via constituent reversal, resulting in the alignment of the focused word with the nuclear prominence phrase-finally (p.15). This duality of strategies available for focus marking led us to further investigate the integration of constituent order with prosodic cues during prominence identification and focus assignment.

In the present study, notable differences emerged when comparing the effects of constituent order in bilingual and monolingual groups during both prominence identification and focus assignment. While monolingual participants were more likely to identify the nuclear accented noun as prominent under the baseline SVO order, bilinguals did not display such a preference, despite the overlap in the baseline order in the dominant grammar and the TL grammar.

Notwithstanding the lower rates of perceived nuclear prominence obtained in the first listening task, we anticipated more accurate performance in the focus assignment task to be achieved via integration of the constituent order with the prosodic expression in the test sentences. Critically, this prediction was confirmed in the data from monolingual Russian speakers, who were more likely to treat the sentence-final nominal constituent as focal in either constituent order, viewing constituent order as a means of focus expression. In contrast, our bilingual participants consistently preferred assigning focus to the nuclear accented noun in the SVO order alone.

These results support recent research on Russian L2 learners and heritage speakers (e.g., Ionin and Luchkina, 2019), which documented a common tendency toward non-target-like interpretations of non-canonical OVS sentences by both adult L2 learners and heritage speakers of Russian. In a similar vein, Ionin et al. (2023a) and Laleko (2022) observed lower overall acceptability of OV[S]_F_ sentences compared to the baseline SV[O]_F_ order, even in the presence of felicitous discourse context.

To date, the sole published investigation addressing the acquisition of Russian focus using methods comparable to ours is Ionin et al. (2023b). Ionin and colleagues tested listeners’ identification of nuclear prominence in relation to contrastive focus in Russian. This study reported highly successful, native-like performance by English-dominant Russian bilinguals, likely attributed to the distinct nature of the nuclear pitch accent marking contrastive focus in Russian. Critically, in Ionin et al. (2023b), both early and late English-Russian bilinguals demonstrated native-like acceptability and perception of Russian contrastive focus prosody tested under variable constituent orders.

In contrast to Ionin et al. (2023b) findings, our bilingual participants demonstrated dissimilar focus assignment preferences when compared to the baseline monolingual Russian speakers. Specifically, when the order of nominal constituents in the test sentences was reversed, bilinguals were notably less inclined to treat the nuclear-accented noun as focus. This suggests that under a non-canonical constituent order, bilinguals differ in their focus assignment preferences from the baseline speakers who clearly identify the nuclear-accented noun as focus, across the tested constituent orders. Further evidence supporting the non-native-like performance of the bilingual speakers was observed through the influence of the TL proficiency observed in both experimental tasks. Specifically, better performance on the cloze deletion test predicted a higher likelihood of selecting the nuclear-accented noun as prominent in Experiment 1, as well as the focus exponent in Experiment 2.

The divergent perception patterns observed in our bilingual speakers’ lend support to the Interface Hypothesis (Sorace and Filiaci, 2006), which predicts non-target-like acquisition patterns specifically at external interfaces. Sorace and Serratrice (2009) further discuss several factors that contribute to the vulnerability of interface phenomena. These include underspecification of interface conditions in the native grammar, cross-linguistic influence, target language (TL) input (quality and quantity), and processing limitations. While the present study does not measure TL input or the processing resources required for focus assignment, the vulnerability in acquiring the Russian focus structure by adult English-Russian bilinguals may be attributed to the unique role of constituent order in signaling information structure and its linkage with phrasal prosody in Russian. Since neither of these properties are present in the L1 grammar, they remain underspecified and thus a potential source of cross-linguistic influence. Additionally, the three-way nature of the interface in question introduces greater inherent complexity, as it involves integrating prosodic cues indicative of the new information status of a discourse referent with constituent order during discourse processing, potentially exacerbating non-target-like performance.

Our approach to participant inclusion on the basis of a cut off TL proficiency score does not enable us to assess if the non-target-like performance observed in the present sample would generalize to speakers with even higher Russian proficiency and/or exceptionally early naturalistic exposure to the TL, similar to studies conducted by Laleko (2022) and Ionin et al. (2023c). The study by Ionin et al. (2023c) found that bilinguals with early naturalistic exposure to Russian in a heritage setting were more native-like in accepting the OV[S]_F_ order in response to narrow subject focus in comparison to late L2ers. Additionally, early bilinguals tested in Ionin et al. were more likely to accept the OVS order as their TL proficiency increased. This trend was not observed among late learners, regardless of their proficiency levels. Laleko (2022), similarly, discovered that English-Russian bilinguals representing diverse backgrounds and proficiency levels generally exhibited lower acceptability of the OVS order, except for high-proficiency heritage speakers who displayed greater acceptability of transitive OV[S]_F_ sentences. While bilinguals in the present study were chosen based on their TL proficiency rather than their age of exposure to Russian (due to an imbalanced number of speakers with early vs. late AOEs), it is conceivable that a more on target performance could emerge in a homogeneous sample of high-performing listeners with particularly early ages of exposure to the TL.

## 6 Conclusion

This study offers new, data-driven insights into the acquisition of the relationship between prosody, constituent order, and information structure in Russian. Our experimental methodology systematically compares how new information focus is signaled in participants’ dominant language (English) and the TL (Russian).

We reported that, in the absence of discourse context, the nuclear pitch accent aligned with the phrase-final nominal acts as a probabilistic, rather than deterministic, indicator for a prosodically prominent reading of the accented word. Further supporting this finding, the evidence of acoustic-prosodic augmentation in relation to the nuclear pitch accenting was subtle in the production data of our model speakers, across tested languages.

As we investigated the link between sentence-final nuclear prominence in Russian and the focal interpretation of clause-final nominal constituents, we discovered notable differences between Russian monolingual speakers and English-Russian bilinguals. Unlike bilinguals, Russian monolinguals exhibited a stronger expectation for phrase-final nuclear prominence in the SVO constituent order compared to the reversed OVS order. Conversely, during focus assignment, English-dominant bilinguals were inclined to assign new information focus to the nuclear accented nominal in the SVO order, and less so - in the subject-final OVS order. In contrast, Russian monolinguals’ preference to assign focus to the nuclear accented nominal upheld irrespective of the constituent order.

Varying performance patterns among baseline Russian speakers and English-Russian bilinguals reveal two key findings: (1) there are no clear a priori expectations about where the main phrasal prominence will occur within an utterance in either English or Russian, and (2) there is less certainty in integrating non-canonical constituent order with phrasal prosody during focus assignment in Russian. This uncertainty leads bilingual listeners to consider both ex-situ elements—the sentence-initial object and the sentence-final subject—as plausible focus exponents.

These findings are largely in line with the IH, which predicts acquisition difficulties for language external interfaces (Sorace and Filiaci, 2006; Montrul and Polinsky, 2011). However, they also highlight the need for testing additional samples of bilingual speakers with earlier ages of target language exposure in a naturalistic setting, as prompted by recent work by Laleko (2022) and Ionin et al. (2023c). Additional research with monolingual Russian speakers is also warranted, to further explore the division of labor between constituent order, prosodic expression, and information structural distinctions in focus marking in Russian.

## Data Availability

The raw data supporting the conclusions of this article will be made available by the authors, without undue reservation.

## Footnotes

^1^The question in (1a) can also receive a response in SVO order, in which case the nuclear pitch accent would be on the preverbal subject; in principle, (2a) can receive a response in OVS order, with the nuclear pitch accent on the preverbal object, though this is rather less commonly attested [see Kallestinova (2007)]. We leave this aside, since the studies reported in this paper examined exclusively sentences with nuclear pitch accent on the sentence-final constituent, as in (1b) and (2b).

^2^In line with Calhoun (2010), Katz and Selkirk (2011) treat both H* and LH* as accents marking new information focus in English.

^3^The focus distinctions in question arise due to distinctions in focus breadth. Broad focus renders two or more adjacent words focal in a phrase or an utterance. Narrow focus assumes the focal status of a single word in a sentence or phrase.

^4^Breen et al. (2010) tested various focus conditions, including broad vs. narrow focus, phrase-final vs. phrase-medial narrow focus, and focus type (contrastive vs. non-contrastive). Only the results relevant for the present study are summarized.

^5^Pitch excursion, measured in semitones, quantifies the degree of nuclear rise in the vicinity of the accented word.

^6^Gussenhoven and Rietveld (1988) point out that the magnitude of pitch excursion due to accenting is affected by phrasal position of the accented words. As a result, accents occurring later in the phrase exhibit smaller excursions than those occurring earlier.

^7^For ease of comparison and in line with prior literature on Russian, our analyses focus exclusively on known tonal correlates of nuclear pitch prominence when modeling the experimental data. Two non-tonal acoustic measures—vocalic segment duration and intensity—were analyzed in the Russian production data. Previous research on Russian [see Luchkina and Cole (2021)] has shown that lower vowel intensity combined with greater segment duration often occurs in the vicinity of the nuclear accented word, likely due to its proximity to the phrase-final boundary. However, in our inferential analyses of perceived word-level prominence and focus assignment, neither intensity nor duration achieved statistical significance. Because a detailed interpretation of these results is beyond the scope of the present study, these measures were excluded from the statistical models.

^8^An anonymous reviewer points out the metalinguistic nature of the prominence identification task and the following focus assignment task (exp. 2) rendering this aspect of the methodology a limitation of the present study. The authors acknowledge the explicit nature of the tasks, deemed necessary to (1) determine the linguistic underpinnings of word-level prominence and (2) the link between perceived word-level prosodic expression and sentence focus. From the methodological standpoint, prosodic analysis by linguistically naïve listeners is used to draw inferences about the underlying prosodic grammar in the so-called rapid prosody transcription tasks (Cole and Shattuck-Hufnagel, 2016; Cole et al., 2019; Bishop et al., 2020, among many others). The backward focus assignment tested in Exp. 2 presents a simplified version of a focus assignment task originally developed for English by Breen et al. (2010). Arguably, the methods chosen for the present study present straightforward, replicable means of testing auditory perception as well as integration of phrasal prosody and constituent order with the extra-linguistic domain of information structure.
