# Erratum: Acquisition of non-contrastive focus in Russian by adult English-dominant bilinguals

**DOI:** 10.3389/fpsyg.2025.1605585

**Published:** 2025-04-11

**Authors:** Frontiers Production Office

**Affiliations:** Frontiers Media SA, Lausanne, Switzerland

**Keywords:** focus, information structure, prosody, constituent order, Russian

Due to a production error, several corrections requested by the author during proofing were not implemented in the final published article. These mainly involved modifications such as rephrasing and word substitution.

The publisher apologizes for this mistake and states that this does not change the scientific conclusions of the article in any way. The original article has been updated.

