# Supplementary material for: Acquisition of non-contrastive focus in Russian by adult English-dominant bilinguals
Source: Front Psychol. 2024 Dec 24;15:1363980. doi: 10.3389/fpsyg.2024.1363980 (PMC11704886; doi:10.3389/fpsyg.2024.1363980)
Supplement: Supplementary file 1 [file Table_1.DOCX]

**Appendix:**

| Variable | | Coefficient | SE | *z* | *p* |
| --- | --- | --- | --- | --- | --- |
| pitch measures | minf0 (subjects) | -.83 | .14 | -5.58 | .0001 |
|  | maxf0  (subjects) | .22 | .04 | 4.97 | .0001 |
|  | f0 excursion  (subjects) | -4.6 | .92 | -5.0 | .0001 |
|  | minf0  (verb) | -.69 | .15 | -4.63 | .0001 |
|  | maxf0  (verb) | .16 | .05 | 3.62 | .0001 |
|  | f0 excursion  (verb) | -3.38 | .92 | -3.66 | .0001 |

| Random-effects Parameters: | Estimate | SE | 95% Conf. Interval |
| --- | --- | --- | --- |
| Participant: | 9.85 | 3.63 | 7.12-.001 |
| Item: | .27 | .05 | .19-.39 |
| sd (Residual) | .87 | .03 | .83-.93 |

Model 1. *Dependent measure: the rate of perceived nuclear prominence in the English monolinguals’ data. Fixed effects: pitch minima, pitch maxima, and pitch excursion. The word from which pitch was measured appears in parenthesis. The random effects parameters include participant and test item (sentence). Likelihood Ratio χ2 =87.26 (p<.0001).*

| Variable | | Coefficient | SE | *z* | *p* |
| --- | --- | --- | --- | --- | --- |
| constituent order  (baseline: SVO) | | .74 | .35 | 2.14 | .03 |
| pitch measures | maxf0  (subjects) *SVO | .00 | .00 | .32 | .8 |
|  | f0 excursion  (subjects) *SVO | -.00 | .02 | -.2 | .8 |
|  | maxf0  (objects) *SVO | .03 | .00 | 1.99 | .05 |
|  | f0 excursion  (objects) *SVO | .07 | .03 | 2.19 | .03 |

| Random-effects Parameters: | Estimate | SE | 95% Conf. Interval | |
| --- | --- | --- | --- | --- |
| Participant: | .17 | .03 | .12 | .23 |
| Item: | 1.63e-09 | 7.56e-09 | 1.60e-13 | .00 |
| sd (Residual) | .42 | .01 | .4 | .45 |

Model 2. *Dependent measure: the rate of perceived nuclear prominence in the Russian monolinguals’ data. Fixed effects: constituent order, pitch maxima, and pitch excursion (the word from which pitch was measured appears in parenthesis). All pitch measures are interacted with constituent order. The random effects parameters include participant and test item (sentence). Likelihood Ratio χ2 =94.52 (p<.0001).*

| Variable | | Coefficient | SE | *z* | *p* |
| --- | --- | --- | --- | --- | --- |
| constituent order  (baseline: SVO) | | -.26 | .3 | -.85 | .4 |
| cloze test score | | .57 | .32 | 1.82 | .07 |
| pitch measures | maxf0  (subjects)*SVO | -.002 | .00 | -2.15 | .03 |
|  | f0 excursion  (subjects)*SVO | -.12 | .03 | -4.14 | .0001 |
|  | f0 max  (objects)*SVO | .006 | .00 | 4.24 | .0001 |
|  | f0 excursion  (objects)*SVO | .15 | .03 | 4.72 | <.0001 |

| Random-effects Parameters: | Estimate | SE | 95% Conf. Interval | |
| --- | --- | --- | --- | --- |
| Participant: | .08 | .23 | .0005 | 131.6 |
| Item: | .0006 | .0002 | .0003 | .001 |
| sd (Residual) | .41 | .01 | .38 | .43 |

Model 3. *Dependent measure: the rate of perceived nuclear prominence in the data from the English-Russian bilinguals. Fixed effects: constituent order, cloze deletion score (as an estimate of the TL proficiency), pitch maxima, and pitch excursion. The word from which pitch was measured appears in parenthesis. All pitch measures are interacted with constituent order. The random effects parameters include participant and test item (sentence). Likelihood Ratio χ2 =53.6 (p<.0001).*

| Variable | | Coefficient | SE | *z* | *p* |
| --- | --- | --- | --- | --- | --- |
| L1  (baseline: Russian) | | -.09 | .07 | -1.28 | .2 |
| constituent order  (baseline: SVO) | | -.03 | .23 | -.15 | .9 |
| pitch measures | maxf0  (subjects)*SVO | -.004 | .00 | -4.42 | <.0001 |
|  | f0 excursion  (subjects)*SVO | -.09 | .02 | -4.35 | <.0001 |
|  | f0 max  (objects)*SVO | .006 | .00 | 5.04 | <.0001 |
|  | f0 excursion  (objects)*SVO | .14 | .02 | 5.95 | <.0001 |

| Random-effects Parameters: | Estimate | SE | 95% Conf. Interval | |
| --- | --- | --- | --- | --- |
| Participant: | .00032 | .0002 | .00009 | .001 |
| Item: | .21 | .06 | .13 | .36 |
| sd (Residual) | .4 | .008 | .39 | .42 |

Model 4. *Dependent measure: the rate of perceived nuclear prominence in the Russian*

*prominence identification task. Fixed effects: constituent order, pitch maxima, and pitch excursion. The word from which pitch was measured appears in parenthesis. All pitch measures are interacted with constituent order. The random effects parameters include participant and test item (sentence). Likelihood Ratio χ2 =79.62 (p<.0001).*

| Variable | | Coefficient | SE | *z* | *p* |
| --- | --- | --- | --- | --- | --- |
| constituent order  (baseline: SVO) | | .68 | .55 | 1.24 | .22 |
| pitch measures | maxf0  (subjects)*SVO | -.00 | .00 | -.5 | .62 |
|  | f0 excursion  (subjects)*SVO | -.01 | .01 | -.6 | .55 |
|  | f0 max  (objects)*SVO | .03 | .02 | 1.87 | .06 |
|  | f0 excursion  (objects)*SVO | .06 | .03 | 2.27 | .02 |

| Random-effects Parameters: | Estimate | SE | 95% Conf. Interval | |
| --- | --- | --- | --- | --- |
| Participant: | .00016 | .0005 | 3.07e | .09 |
| Item: | .16 | .1 | .04 | .57 |
| sd (Residual) | .42 | .01 | .4 | .45 |

Model 5. *Dependent measure: the rate of focus assignment to the nuclear accented noun by Russian monolinguals. Fixed effects: constituent order, pitch maxima, and pitch excursion. The word from which pitch was measured appears in parenthesis. All pitch measures are interacted with constituent order. The random effects parameters include participant and test item (sentence). Likelihood Ratio χ2 =21.6 (p=.01).*

| Variable | | Coefficient | SE | *z* | *p* |
| --- | --- | --- | --- | --- | --- |
| constituent order  (baseline: SVO) | | .62 | .31 | 1.97 | .05 |
| cloze test score | | .75 | .3 | 2.5 | .01 |
| pitch measures | max f0  (subjects)*SVO | -.003 | .001 | -1.99 | .05 |
|  | f0 excursion  (subjects)*SVO | -.05 | .03 | -1.48 | .14 |
|  | f0 max  (objects)*SVO | -.00 | .002 | -.03 | .97 |
|  | f0 excursion  (objects)*SVO | .007 | .03 | .21 | .84 |

| Random-effects Parameters: | Estimate | SE | 95% Conf. Interval | |
| --- | --- | --- | --- | --- |
| Participant: | 1.26e-07 | 4.98e-07 | 5.37e-11 | .0003 |
| Item: | .23 | .04 | .17 | .32 |
| sd (Residual) | .41 | .01 | .39 | .44 |

Model 6. *Dependent measure: the rate of focus assignment to the nuclear accented noun perceived by Russian monolinguals. Fixed effects: constituent order, pitch maxima, and pitch excursion. The word from which pitch was measured appears in parenthesis. All pitch measures are interacted with constituent order. The random effects parameters include participant and test item (sentence). Likelihood Ratio χ2 =23 (p=.03).*

| Variable | | Coefficient | SE | *z* | *p* |
| --- | --- | --- | --- | --- | --- |
| L1  (baseline: Russian)  constituent order  (baseline: SVO) | | .14 | .06 | 2.22 | .03 |
|  |  | 1.2 | .67 | 1.8 | .07 |
| pitch measures | maxima,  subjects*SVO | -.001 | .001 | -0.86 | 0.39 |
|  | excursion,  subjects*SVO | -.05 | .03 | -2.35 | .02 |
|  | maxima, objects*SVO | .004 | .002 | 2.16 | .03 |
|  | excursion,  objects*SVO | .05 | .03 | 1.95 | .05 |

| Random-effects Parameters: | Estimate | SE | 95% Conf. Interval | |
| --- | --- | --- | --- | --- |
| Participant: | .00007 | .001 | 1.57e-21 | 2.85e+12 |
| Item: | .17 | .1 | .05 | .54 |
| sd (Residual) | .42 | .012 | .4 | .44 |

Model 7. *Dependent measure: the rate of focus assignment to the nuclear accented noun by all focus assignment task participants. Fixed effects: constituent order, pitch maxima, and pitch excursion. The word from which pitch was measured appears in parenthesis. All pitch measures are interacted with constituent order. The random effects parameters include participant and test item (sentence). Likelihood Ratio χ2 =32.99 (p=.001).*
